# Spring maize yield, soil water use and water use efficiency under plastic film and straw mulches in the Loess Plateau

**DOI:** 10.1038/srep38995

**Published:** 2016-12-15

**Authors:** Wen Lin, Wenzhao Liu, Qingwu Xue

**Affiliations:** 1 State Key Laboratory of Soil Erosion and Dryland Farming on the Loess Plateau, Institute of Soil and Water Conservation, Chinese Academy of Sciences and Ministry of Water Resources, Yangling, Shaanxi 712100, China; 2 University of Chinese Academy of Sciences, Beijing 100049, China; 3 Institute of Soil and Water Conservation, Northwest A&F University, Yangling 712100, China; 4 Texas A&M AgriLife Research and Extension Center, 6500 Amarillo Blvd. W., Amarillo, Texas 79106, USA

## Abstract

To compare the soil water balance, yield and water use efficiency (WUE) of spring maize under different mulching types in the Loess Plateau, a 7-year field experiment was conducted in the Changwu region of the Loess Plateau. Three treatments were used in this experiment: straw mulch (SM), plastic film mulch (PM) and conventional covering without mulch (CK). Results show that the soil water change of dryland spring maize was as deep as 300 cm depth and hence 300 cm is recommended as the minimum depth when measure the soil water in this region. Water use (ET) did not differ significantly among the treatments. However, grain yield was significantly higher in PM compared with CK. WUE was significantly higher in PM than in CK for most years of the experiment. Although ET tended to be higher in PM than in the other treatments (without significance), the evaporation of water in the fallow period also decreased. Thus, PM is sustainable with respect to soil water balance. The 7-year experiment and the supplemental experiment thus confirmed that straw mulching at the seedling stage may lead to yield reduction and this effect can be mitigated by delaying the straw application to three-leaf stage.

Water scarcity is the main limiting factor for agriculture production in dryland farming systems^1^. In dryland farming areas, precipitation is low and unevenly distributed, whereas potential evapotranspiration is high. Hence, the limited water resources cannot meet the requirements for crop growth, and yields are low compared with those of humid and semi-humid regions^2^. Dryland farming systems are typical in most of the Loess Plateau, where the limited and uneven distribution of precipitation is a great threat to crop yield.

Crop production under dryland is largely related to soil water from both growing season and fallow period. Soil water evaporation, particularly during fallow and early growing season, can significantly reduce soil water availability to plant growth. One method of reducing soil evaporation to increase soil water is to cover the soil surface^3^. In the Loess Plateau, straw mulching and plastic film mulching are two common mulching practices that are widely used in crop production, particularly maize production. Straw mulching reduces soil water evaporation^4^ and increases water use efficiency (WUE) and crop yield^4^,^5^,^6^,^7^. Straw mulching also affects soil temperature^4^,^8^ by increasing soil temperature in winter and decreasing soil temperature at other times of the year^4^. For spring maize in the Loess Plateau, low soil temperature at the early growing stage may constrain crop growth and lead to yield reduction^9^,^10^,^11^,^12^,^13^. Plastic film is another mulching material that is widely applied in agriculture production in the Loess Plateau^14^. Plastic film mulching can reduce soil water evaporation and hence increase soil moisture for crop growth^15^. Moreover, in the Loess Plateau, spring maize is sown in late April, when the soil temperature is still low. Numerous studies have demonstrated that plastic film mulching can increase soil surface temperature^11^,^15^,^16^,^17^,^18^. Hence, the warming effect of plastic film mulch is helpful for the growth of maize in spring. The increased temperature in the surface soil under plastic film mulching accelerates phenology compared with no mulching^18^. Higher soil moisture and soil temperature can promote root development^19^, thus enhancing water absorption capacity^8^. The favourable temperature and moisture conditions under plastic film mulching also enhance plant nutrient uptake^20^. Thus, yield and WUE are higher under plastic film mulching. However, potential hazards of plastic film mulching include accelerated decomposition of soil organic matter and rapid depletion of soil fertility^21^. and soil water^9^; thus, plastic film mulching may fail to sustain crop production in the long run.

Although there are many studies on plastic film mulch, most of these studies were short-term (2–3 years). Since climate conditions, particularly precipitation, differ greatly among years in the Loess Plateau, short-term field experiments are not sufficiently representative of long-term climatic conditions. Some studies showed the benefits of soil water conservation and increasing yield for using straw mulching. However, there still are risks for reducing yield using straw mulching in spring maize. In this study, we report the results of a 7-year field experiment in the Loess Plateau. The primary objective was to evaluate the soil water sustainability of plastic film mulching. In addition, we also explored the reason of yield reduction effect of straw mulch in the south Loess Plateau.

## Results

### Soil water depletion during growing season

In the 0–300 cm soil layer, after 7 years, the soil water storage decreased only by 8 mm in PM, a smaller change than in SM (22 mm) and CK (55 mm) (Table 1). In the 0–600 cm soil profile, the soil water did not change for SM and PM but slightly reduced by 44 mm. The results also shown that soil water depletion only occurred in dry years (seasonal precipitation of less than 400 mm), and the depletion depth was primarily in the 0–300 cm profile. In relatively wet years (2010, 2011 and 2013), the soil water was not depleted but increased (Table 1).

Compared with the other 4 years, soil water depletion was markedly different in 2010, 2011 and 2013. Instead of depletion of water by crop system evapotranspiration, the soil water in the upper soil layer (from surface to 200–400 cm depth) increased during these 3 years. The wetting front was approximately 400, 250 and 200 cm, respectively. Precipitation in 2010, 2011 and 2013 was 543, 468 and 400 cm, respectively, much higher than that in 2009, 2010 and 2014, when the respective seasonal precipitation was 357, 344 and 268 mm (Table 1). The higher the seasonal precipitation, the deeper the wetting front. Moreover, the wetting fronts differed among the treatments, i.e., PM ＜ CK ＜ SM.

**Figure 1:**
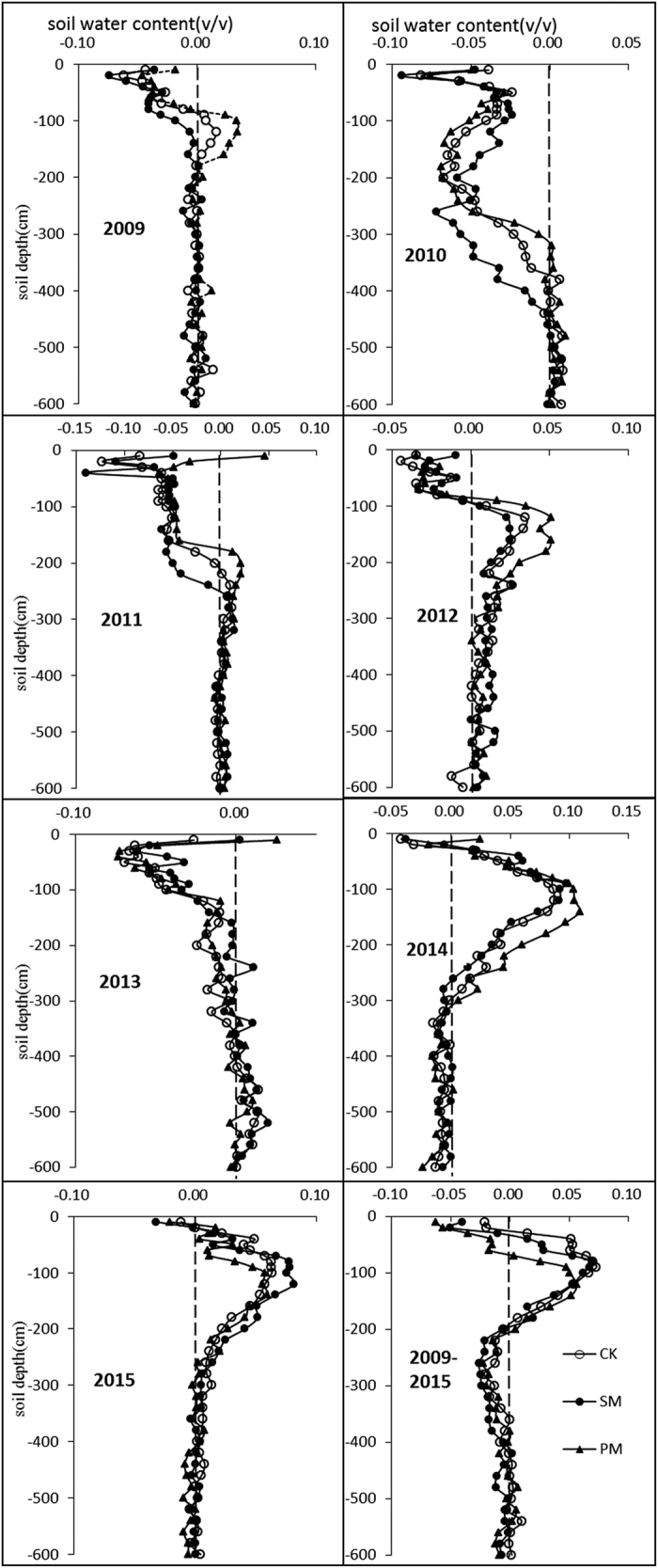
Soil water depletion in the 0–600 cm profile. The depletion rate was calculated by subtracting the soil water content before sowing from the soil water content (v/v) after harvesting. The value in the last sub-fig is SWC at harvest time in 2015 minus SWC before sowing in 2009.

The soil water depletion depth during maize growing season varied from year to year (Fig. 1). In 2009, soil water depletion occurred at a depth of 100–180 cm for PM and CK but soil water in the 0–100 cm soil profile did not decrease but increased at harvesting. No soil water depletion was observed for SM and soil water increased in the 0–180 cm soil profile at harvesting. For the entire 0–600 cm soil profile, soil water storage increased by 27 and 55 mm for CK and SM, respectively, but remained stable for PM (Table 1). Similar results were obtained in 2012. For all treatments, soil water depletion occurred at the 100–300 cm soil depth but increased in the 0–100 cm soil layer. The overall soil profile exhibited a depletion trend, with depletion of 34, 49 and 65 mm, respectively, for CK, SM and PM. Compared with 2009, the depletion phenomena in 2012 occurred at greater depths, and deep soil water depletion (300–600 cm) accounted for 38%, 67% and 36% of the total depletion. In 2014, depletion occurred nearly entire 0–300 cm soil profile, except for a slight increase in soil water in the 0–20 cm soil layer. The pattern of soil water depletion in 2015 was the same as that in 2014, except that the depletion depth was approximately 220 cm. For all the soil layers with water depletion, the amount of water depletion was greater for PM than for the other two treatments, but no significant difference between CK and SM was observed. For soil layers deeper than 300 cm, soil water remained nearly unchanged after maize growth. After 7 spring maize growing seasons, the soil water depletion mainly occurred at 0–200 cm profile. From 200 cm to 400 cm, the soil water increased to some extent. The overall trend was similar for the three different treatments. Specifically, more soil water in the 100–200 cm profile was consumed in PM than in the other two treatments.

### Soil water balance during the fallow period

The fallow period refers to the time between the harvest date of the previous crop and the sowing date of the current crop, e.g., the fallow period of 2010 refers to the time between mid-September 2009 and mid-April 2010. The growing season of spring maize in the Loess Plateau is only approximately 5 months from April to August. Thus, the fallow time for the region’s spring maize system is approximately 7 months. During these 7 months, there is no crop in the field, and the soil surface is bare (CK) or has only the residual plastic film (PM) or straw (SM). The film and straw can still reduce evaporation, but the effect is limited because the film is broken and the straw is partially decomposed. After the rainy season has passed, soil water evaporation continues. Han *et al*.^22^ reported that the potential evapotranspiration (ET0) from late September to late April is 348 mm, accounting for approximately 36.7% of the yearly ET_0_. Thus, the soil water change during this period for the spring maize growing system cannot be ignored.

**Figure 2:**
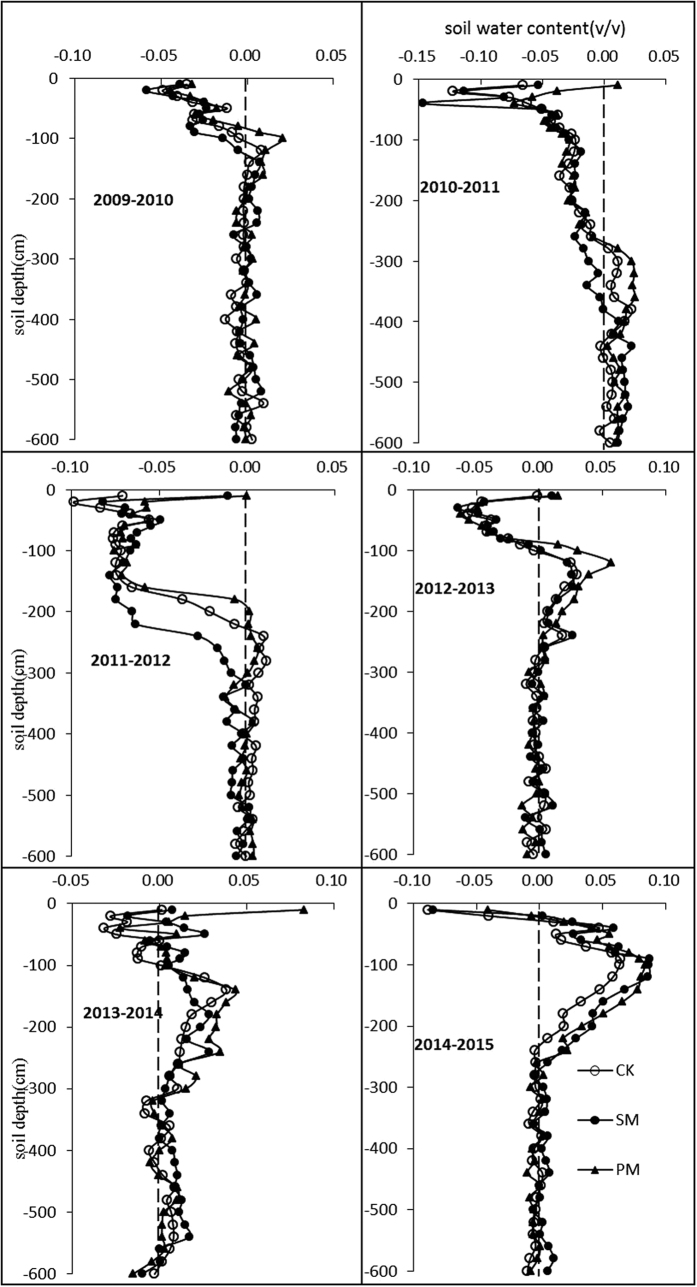
Soil water change in the 0–600 cm soil layer in the fallow period. The change rate was calculated by subtracting the soil water content after the previous harvest from the soil water content (v/v) before sowing.

For most of the years, even without crop water uptake, soil water still decreased after the fallow season. In the 0–600 cm soil layer, the soil water storage decreased, except in 2014 and 2015 (Table 2). As shown in Fig. 2, the water depleted in winter was mainly that in the upper 0–100 cm soil profile. The soil water in the 0–600 cm profile increased in 2014 and 2015, primarily due to precipitation in the fallow period. In 2014 and 2015, the precipitation in the fallow period was 238 mm and 227 mm, much higher than that in the fallow periods of 2010, 2011 and 2013, when the precipitation was only approximately 100 mm. In addition to the amount of precipitation, the precipitation distribution during the fallow period also affected soil water content before planting. For example, the fallow-period precipitation was similar in 2012, 2014 and 2015, but the soil water changes were different. The water storage in the 0–600 cm soil profile increased in 2014 and 2015 but decreased in 2012. From the precipitation distribution, precipitation mainly occurred in autumn and early winter of 2011 (217 mm) and was only 28 mm for the early spring in 2012 before planting. A great amount of precipitation after harvest in 2011 wetted the whole soil profile, but subsequent precipitation before the next planting was low. Consequently, much water evaporated in the upper soil layer. For 2014 and 2015, the precipitation in the previous and current early spring was high (both 119 mm in 2014; 132 and 95 mm in 2015). The precipitation in the previous autumn was not sufficient to wet the whole profile, and the wet front only reached to 300 cm. More precipitation in early spring led to more water storage compared with 2012.

Although the plastic film degraded during the fallow season, water conservation was superior in PM. With PM mulching, soil water decreased less in dry years but increased more in wet years. There was no significant water conservation effect of straw mulch in the winter fallow season.

### Yield, Water Use (ET) and Water Use Efficiency

Although the maize yield varied among growing seasons, the yield was significantly higher for PM than for the other two treatments (Table 3). In 2009 and 2011, the yield was significantly lower for SM than for CK. Yield was also lower in SM in 2010, but the difference was not significant. Based on the results for the first 3 years, SM reduced the crop yield, possibly due to the lower temperature under SM in the early growth stage. Consequently, from 2012 onward, we adjusted the application time for straw mulching. The yield difference between CK and SM subsequently reversed, and yield was significantly higher for SM than for CK, except in 2014. In 2009 and 2011, ET was significantly higher under PM than under CK and SM. However, in later years, except 2013, there were no significant differences in ET among treatments even.

Compared with CK, PM significantly increased WUE in all years except 2009. The WUE for PM in 2011, 2013, 2014 and 2015 also increased significantly compared with that for SM. There was no difference in WUE between SM and CK.

**Figure 3:**
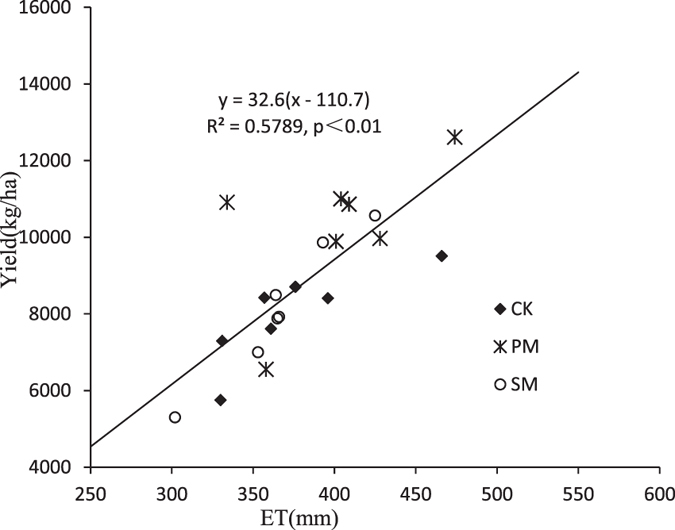
Yield-ET relationship for different treatments. The straight line is a fit line for the data of SM and CK.

There was a linear relationship between yield and ET after pooling data from different years and treatments (Fig. 3). For PM, 6 of the 7 data points were at the upper side of the fitted regression line, indicating that the WUE of PM was higher than that of the other treatments in most years. The relationship between maize yields and seasonal ET can be described by a linear function y = 32.6(x − 110.7), in the Changwu region. From the study of Zhang^14^, the attainable maize yield can be expressed by a boundary function for maize in the Loess plateau y = 40(x − 40). There still a wide gap in attainable water use efficiency and minimum soil evaporation for us to attain.

### Effect of straw application timing on yield

The supplemental experiments demonstrated that compared with no mulching, straw mulching at seedling stage reduced maize yield. whereas straw mulching at three-leaf stage increased the grain yield (Table 4). In this supplemental experiment, the only variable was straw application timing. Hence straw mulch in the three-leaf stage can increase the maize yield seedling.

## Discussion

### Soil water use of spring maize

In the Loess Plateau, spring maize growth season coincides with the wet season. Thus, precipitation during growing season is important to maize yield^6^. In wet years, precipitation exceeded the crop requirement and the soil water storage at harvest time was higher than that during sowing time (2009–2011 and 2013). In dry years, soil water was used by crops (2012, 2014 and 2015). The maize rooting depth in the Loess Plateau is less than 200 cm and previous studies mainly used 200 cm as the water cycle depth^23^,^24^ as it is thought that 90% of ET normally comes from this zone^25^. In our study, the soil water mainly changed in the 0–300 cm soil profile, at depths below 300 cm, it remained nearly constant, with the exception of 2010 when the precipitation during the maize growing season was 543 mm, and the water recharged the soil down to 400 cm. Hence, after 7 years of maize growth in our research, the soil water from 0–200 cm was reduced, mainly due to the depletion in the last year rather than a long-term effect. Thus, 200 cm as the soil water measure depth is not precise in ET estimation in this region: in wet years, the rainwater may infiltrated exceed 200 cm and the ET will be overestimated; while in dry years, the water under 200 cm may be used and ET will be under estimated. So the recommend soil water measure depth should not less than 300 cm.

### Plastic film mulch and soil water sustainability

Plastic film mulch increased soil temperature and reduced soil evaporation^9^,^18^, which It is critical for improving the grain yield of spring maize in the Loess Plateau^16^,^26^. In our 7 successive years of experiment, the yield varied greatly among years due to the climatic variability, particularly the precipitation. The yield for PM was significantly higher than that of SM and CK in all years. However, due to increased plant growth and higher plant T rates, plant under plastic film mulch requires higher water input^9^,^10^,^17^,^18^. Hence plastic mulch systems may not be sustainable because plants under plastic mulch used soil nutrients and water more quickly^9^. In our study, ET for PM was higher than that of CK only in 2 years (Tables 1 and 3). PM practices reduced the ratio of soil evaporation and maize transpiration^8^,^14^. Moreover, plastic film mulching would lead to an increased root volume^23^. Long-term additions of organic materials to the soil should increase the soil water-holding capacity^27^, hence more water was recharged for PM during the subsequent fallow period was maintained in our research (Table 2). After 7 years of cultivation, the soil water storage under PM did not change (Table 1). Thus, our results indicated that PM is sustainable with respect to soil water, which is disagree with the previous studies in this region^9^. The reason for a inconsistent conclusions with previous studies is our results are based on a 7-year field experiment. The results of 2–3 years experiment are prone to be influenced by short-term climatic conditions and not representative to explain the lone term effect. Our previous study in this region showed that soil water storage within the 300 cm depth would be fully recharged once in less than 10 years under cropping systems other than wheat^25^. Hence short term field result is not enough to explain the long term effect.

### Yield effect of straw mulch

Straw mulching is controversial in the Loess Plateau. Maize straw mulching can prevent water evaporation and improve crop yield and WUE^17^,^28^. However, straw mulching reduces soil temperature during sowing and the early stage of maize development. Thus, instead of enhancing crop yield, straw mulch may cause yield reduction^9^,^11^. In our study, crop yield under SM was lower than that under CK before 2011 and higher after 2012. The maize straw was applied at seedling stage from 2009 to 2011 and at three-leaf stage from 2012 to 2015. In a study by Zhang^9^ conducted in the same area from 2007 to 2009, a higher yield was obtained under SM in 2007, whereas a lower yield was obtained in 2008 and 2009. In 2007, the straw was applied at three-leaf stage, whereas in the next two years, it was applied at seedling stage. Zhang suggested that delaying straw mulching application had minor negative effects on soil temperature at early stages but conserved more water later, hence stimulating maize growth. The results of our study agreed with those from Zhang. Supplemental experiment confirmed that the yield effect of straw mulch depends on the straw application timing (Table 4), lower temperature in the early stage may be part of the reason that impacted the maize growth in the early stage and further lead to yield reduction. From the field experiment, mechanical resistance of straw to the plant may be another reason since the seedlings are fragile. In some places in the Loess Plateau, straw mulch showed a positive influence on spring maize yield. For example, the results in Heyang of Shaanxi province showed that compared with CK, SM increased maize yield significantly^29^. Similar results also found in Chengcheng, another county in Shaanxi province^24^. This is because straw mulching effects also depend on the climatic condition^11^. We noticed that the annual mean temperature in Heyang and Chengcheng is about 1 °C higher than that of Changwu. The lower surface temperature under SM may affect maize growth in cold region and lead to yield reduction.

## Conclusion

The soil water content changed as deep as 300 cm in spring maize field in the Changwu region. Therefore, at least 300 cm is recommended when measure the soil water content in this region. Plastic film mulching consistently increased spring maize yield and WUE in the southern part of the Loess Plateau. However, under PM, soil water depletion was observed at certain depths of the soil during the maize growing seasons, whereas in the fallow period, more water was recharged at these depths. Hence, compared with the SM and CK treatments, no depletion trend was observed for the stored soil water in the 0–600 cm soil layer after 7 years of plastic film application. PM is thus sustainable with respect to soil water. Lower soil temperature and mechanical resistance attributed to the yield reduction effect of spring maize in the Changwu region. Adapting straw mulching at seedling stage to the three-leaf stage may mitigate such disadvantage and increase the crop yield. Compared with PM, the yield-increasing effect of SM is much lower and unstable. Additionally, although the remaining plastic film is removed from the soil before sowing the next crop, a small amount of residual film is unavoidable. To be truly environmentally friendly and sustainable, degradable mulching film is advised for local farming.

## Materials and Methods

### Site description

**Figure 4:**
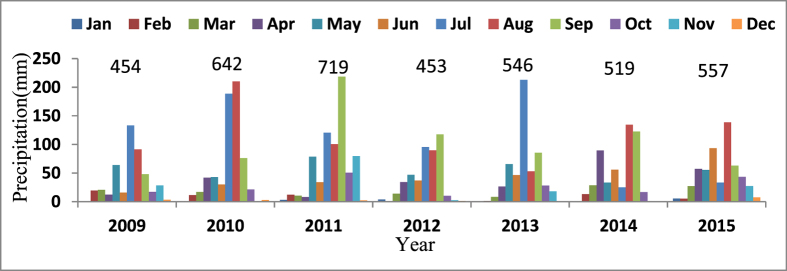
Precipitation distribution for the 7 years of this study. The figures above the bars are the annual total precipitation.

The study was conducted in Changwu county, Shaanxi province, China (35.14N, 107.41E and 1206 m above sea level) from 2009 to 2015. Changwu county is located in the warm temperate zone and has a continental monsoon climate. The precipitation in the Loess Plateau is uneven (Fig. 4). During the 7 years of the experiment, the annual precipitation varied from 453 mm to 719 mm. The growing season precipitation from April to September varied from 421 mm to 589 mm, representing 78%–93% of the annual precipitation. The annual temperature is 9.1 °C. The soil is light silt loam (Heilutu series). The experimental site was located on the flat tableland, where the groundwater table is 60 m below the soil surface.

## Experimental Design

### Experiment 1

The experiment was conducted from 2009 to 2015. Three treatments were used in this experiment: (1) conventional practice without soil cover (CK), (2) straw mulch with 9000 kg/ha maize straw (SM) and (3) plastic film mulch (PM). The PM was covered before sowing. The experiment was a randomized completely block design with three replications. The plot size was 10.3 m long and 6.5 m wide. Based on soil testing, fertilizers (135 kg/ha N and 90 kg/ha P_2_O_5_) were spread over the soil surface and incorporated into the 0–20 cm soil layer using a rotary cultivator before sowing. Maize was sown with a row space of 60 cm and at a density of 56,000 seeds/ha. The sowing date and harvest date are shown in Table 5.

At harvest time, the maize was harvested manually.All grains in each plot were collected and air dried to determine the crop yield (15.5% moisture). After harvest, all aboveground parts of the maize were removed, leaving the remaining plastic film and half-decomposed covering straw in the field. In the subsequent April, the plastic film and the remaining straw were removed (and not used again) to prepare the land for the next crop. During the whole growing season, weeds were hand-weeded. No major insect problems were found each growing season.

### Experiment 2

This experiment was a supplement of experiment 1. From the results of experiment 1, the yield increasing effect of SM after 2012 may be due to the adaption of mulching stage. To clarify this effect, we carried out experiment 2 in 2014. There were three treatments: CK, no straw mulching; T1, straw applied in seedling stage; T2, straw applied in three-leaf stage. All the other field management practices the same as experiment 1.

### Measurements and calculations

The precipitation data were recorded using a standard weather station located near (within 100 m) the experimental site.

The soil water content in 0–600 cm soil profile was measured before sowing and after harvest using a neutron probe. Neutron probe tubes were installed in three replicate plots of each treatment, The neutron probe was calibrated against gravimetrically measured soil moisture contents using soil cores. To ensure the accuracy, the neutron probe is calibrated yearly. For the 0–100 cm soil profile, the measurement was taken at depth intervals of 10 cm; for the 100-600 cm profile, the measurement was taken at depth intervals of 20 cm.

Because the experimental field was flat and the groundwater table was deep, deep percolation and runoff of water in the field can be neglected. Thus, evapotranspiration (ET), soil water depletion in maize growing stage (ΔW) and soil water recharge in fallow period (ΔSW) were determined using the formulas:


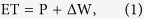



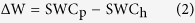



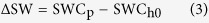


where P is the precipitation during the whole growing season, SWC_h_ is soil water content in harvest time, SWC_p_ is soil water content before planting, SWC_h0_ is soil water content at the previous harvest time.

Water use efficiency (WUE) was calculated using the formula (4), where Y is the grain yield.


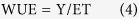


### Statistical analysis

The analysis of variance was conducted using SAS 9.3 with appropriate experimental design to detect main effects of year and mulching and their interactions. Least significant difference (LSD) was used to conduct mean comparison at P < 0.05.

## Additional Information

**How to cite this article**: Lin, W. *et al*. Spring maize yield, soil water use and water use efficiency under plastic film and straw mulches in the Loess Plateau. *Sci. Rep.* **6**, 38995; doi: 10.1038/srep38995 (2016).

**Publisher’s note:** Springer Nature remains neutral with regard to jurisdictional claims in published maps and institutional affiliations.

## Figures and Tables

**Table 1:** Soil water depletion in the 6-m profile (mm).

Treatment	Precipitation mm	0–600 cm			0–300 cm		
		CK	SM	PM	CK	SM	PM
2009	357	−27ab	−55a	1b	−26ab	−50a	−2b
2010	543	−144b	−177a	−142b	−143a	−143a	−151a
2011	468	−107a	−115a	−40b	−103a	−126a	−47b
2012	344	32a	49a	60a	20a	18a	44b
2013	400	−69a	−35a	−66a	−78a	−54a	−70a
2014	268	89a	96a	141a	111a	111a	164b
2015	361	105b	113b	64a	93ab	109b	73a

Values followed by different letters within a row are significantly different (P < 0.05).

**Table 2:** Soil water change during the fallow season (mm) from maize harvesting to next spring before maize planting.

Year	Precipitation mm	0–600 cm			0–300 cm		
		CK	SM	PM	CK	SM	PM
2009–2010	92	−38b	−31b	−15a	−28a	−29a	−13a
2010–2011	95	−68b	−74b	−28a	−89a	−104a	−70a
2011–2012	245	−119a	−180a	−100a	−124ab	−159b	−96a
2012–2013	107	−18a	−5a	−6a	−10b	−2b	11a
2013–2014	238	35a	51a	71a	25a	33a	62a
2014–2015	227	41c	114a	97b	52b	104a	109a

Values followed by different letters within a row are significantly different (P < 0.05).

**Table 3:** Yield, Water Use (ET) and Water Use Efficiency of spring maize under different treatments.

TR	Yr	Yield (kg/ha)	ET (mm)	WUE (kg/ha/mm)
CK	2009	5752b	330b	17.5a
SM		5302c	302ab	17.6a
PM		6556a	358a	18.3a
CK	2010	8410b	396a	21.2b
SM		7925b	366a	21.7ab
PM		9895a	401a	24.7a
CK	2011	7616b	361b	21.1ab
SM		7001c	353b	19.8b
PM		9972a	428a	23.3a
CK	2012	8709c	376a	23.2b
SM		9864b	393a	25.1ab
PM		11005a	404a	27.2a
CK	2013	7299c	331a	22.1b
SM		7882b	365a	21.6b
PM		10914a	334a	32.7a
CK	2014	8423b	357a	23.6b
SM		8494b	364a	23.3b
PM		10864a	409a	26.6a
CK	2015	9513c	466a	20.4b
SM		10566b	474a	22.3b
PM		12615a	425b	29.7a

The lowercase letters following the numbers in each column for each year indicate significant (P < 0.05) differences between treatments based on LSD tests.

**Table 4:** Impact of straw application time on maize yield.

Treatment	Yield (kg/ha)	Biomass (kg/ha)	Yield effect compared with CK (%)
CK	7635b	16175b	—
T1	7092c	16055b	−7.1
T2	8232a	16755a	7.8

CK, no straw mulching; T1, straw applied in seedling stage; T2, straw applied at three-leaf stage.

The yield effect compared with CK indicates the % yield increase or decrease compared with CK.

**Table 5:** Maize variety, sowing date, straw mulch application and harvest date during the seven crop years.

Crop year	Variety	Sowing date	Straw mulch application	Harvest date
2009	Jinsui 9	April 17^th^	Sowing	Sep. 7^th^
2010	Jinsui 9	April 15^th^	Sowing	Sep. 14^th^
2011	Jinsui 9	April 16^th^	Sowing	Sep. 14^th^
2012	Xianyu 335	April 8^th^	Three-leaf	Sep. 9^th^
2013	Xianyu 335	April 8^th^	Three-leaf	Sep. 7^th^
2014	Xianyu 335	April 9^th^	Three-leaf	Sep. 9^th^
2015	Xianyu 335	April 24^th^	Three-leaf	Sep. 15^th^

## References

[b1] Rockström J., Lannerstad M. & Falkenmark M. Assessing the water challenge of a new green revolution in developing countries. Proceedings of the National Academy of Sciences 104, 6253–6260 (2007). 10.1073/pnas.0605739104 PMC1851042 17404216

[b2] Parr J., Stewart B., Hornick S. & Singh R. In Advances in soil science 1–8 (1990).

[b3] Ren X. L., Zhang P., Chen X. L., Guo J. J. & Jia Z. K. Effect of Different Mulches under Rainfall Concentration System on Corn Production in the Semi-arid Areas of the Loess Plateau. Sci Rep 6, 10, doi: 10.1038/srep19019 (2016). 26751619 PMC4707461

[b4] Chen S., Zhang X., Pei D., Sun H. & Chen S. Effects of straw mulching on soil temperature, evaporation and yield of winter wheat: field experiments on the North China Plain. Annals of Applied Biology 150, 261–268 (2007).

[b5] Wang T. C., Wei L., Wang H. Z., Ma S. C. & Ma B. L. Responses of rainwater conservation, precipitation-use efficiency and grain yield of summer maize to a furrow-planting and straw-mulching system in northern China. Field Crop. Res. 124, 223–230 (2011).

[b6] Qin W., Hu C. S. & Oenema O. Soil mulching significantly enhances yields and water and nitrogen use efficiencies of maize and wheat: a meta-analysis. Sci Rep 5, 13, doi: 10.1038/srep16210 (2015). PMC4653642 26586114

[b7] Lal R. Soil temperature, soil moisture and maize yield from mulched and unmulched tropical soils. Plant and Soil 40, 129–143 (1974).

[b8] Li S. X., Wang Z. H., Li S. Q., Gao Y. J. & Tian X. H. Effect of plastic sheet mulch, wheat straw mulch, and maize growth on water loss by evaporation in dryland areas of China. Agricultural water management 116, 39–49 (2013).

[b9] Zhang S. L., Li P. R., Yang X. Y., Wang Z. H. & Chen X. P. Effects of tillage and plastic mulch on soil water, growth and yield of spring-sown maize. Soil & Tillage Research 112, 92–97, doi: 10.1016/j.still.2010.11.006 (2011).

[b10] Liu Y., Shen Y. F., Yang S. J., Li S. Q. & Chen F. Effect of mulch and irrigation practices on soil water, soil temperature and the grain yield of maize (Zea mays L) in Loess Plateau, China. African Journal of Agricultural Research 6, 2175–2182 (2011).

[b11] Gao Y. J. & Li S. X. Cause and mechanism of crop yield reduction under straw mulch in dryland. Transactions Of The Chinese Society of Agricultural Engineering 21, 15–19 (2005).

[b12] Dam R. . Soil bulk density and crop yield under eleven consecutive years of corn with different tillage and residue practices in a sandy loam soil in central Canada. Soil and Tillage Research 84, 41–53 (2005).

[b13] Cao G. F. A study on the Method of Mini-area Cultivation, Vovering Mtarerial and Aupplementary Irrigation Period in Cold and Semi-Arid Area. Agricultural Research In The Arid Areas 16, 13–18 (in Chinese) (1998).

[b14] Zhang S. L., Sadras V., Chen X. & Zhang F. Water use efficiency of dryland maize in the Loess Plateau of China in response to crop management. Field Crop. Res. 163, 55–63 (2014).

[b15] Cook H. F., Valdes G. S. & Lee H. C. Mulch effects on rainfall interception, soil physical characteristics and temperature under Zea mays L. Soil and Tillage Research 91, 227–235 (2006).

[b16] Liu C. A. . Effects of plastic film mulch and tillage on maize productivity and soil parameters. Eur. J. Agron. 31, 241–249, doi: 10.1016/j.eja.2009.08.004 (2009).

[b17] Li R., Hou X. Q., Jia Z. K., Han Q. F. & Yang B. P. Effects of rainfall harvesting and mulching technologies on soil water, temperature, and maize yield in Loess Plateau region of China. Soil Research 50, 105–113, doi: 10.1071/sr11331 (2012).

[b18] Bu L. D. . The effects of mulching on maize growth, yield and water use in a semi-arid region. Agricultural Water Management 123, 71–78 (2013).

[b19] Cumbus I. & Nye P. Root zone temperature effects on growth and phosphate absorption in rape Brassica napus cv. Emerald. Journal of experimental botany 36, 219–227 (1985).

[b20] Boatwright G., Ferguson H. & Sims J. Soil temperature around the crown node influences early growth, nutrient uptake, and nutrient translocation of spring wheat. Agronomy Journal 68, 227–231 (1976).

[b21] Li Y. S. . Influence of continuous plastic film mulching on yield, water use efficiency and soil properties of rice fields under non-flooding condition. Soil and tillage Research 93, 370–378 (2007).

[b22] Han X. Y., Liu W. Z. & Lin W. Spatiotemporal analysis of potential evapotranspiration in the Changwu tableland from 1957 to 2012. Meteorological Applications 22, 586–591 (2015).

[b23] Gao Y. H., Xie Y. P., Jiang H. Y., Wu B. & Niu J. Y. Soil water status and root distribution across the rooting zone in maize with plastic film mulching. Field Crop. Res. 156, 40–47 (2014).

[b24] Li S. X., Wang Z. H., Li S. Q. & Gao Y. J. Effect of nitrogen fertilization under plastic mulched and non-plastic mulched conditions on water use by maize plants in dryland areas of China. Agricultural Water Management 162, 15–32 (2015).

[b25] Liu W. Z. . Soil water dynamics and deep soil recharge in a record wet year in the southern Loess Plateau of China. Agricultural Water Management 97, 1133–1138 (2010).

[b26] Liu Y., Yang S. J., Li S. Q., Chen X. P. & Chen F. Growth and development of maize (Zea mays L.) in response to different field water management practices: Resource capture and use efficiency. Agricultural and Forest Meteorology 150, 606–613 (2010).

[b27] Wang J., Liu W. Z. & Dang T. H. Responses of soil water balance and precipitation storage efficiency to increased fertilizer application in winter wheat. Plant and soil 347, 41–51 (2011).

[b28] Chen X. L., Wu P. T., Zhao X. N. & Persaud N. Effect of Different Mulches on Harvested Rainfall Use Efficiency for Corn (Zea mays L.) in Semi-Arid Regions of Northwest China. Arid Land Research and Management 27, 272–285, doi: 10.1080/15324982.2013.771231 (2013).

[b29] Cai T. Y. . Effects of different rates of straw mulch on soil moisture and yield of spring maize in Weibei Highland area of China. Transactions of the Chinese Society of Agricultural Engineering 27, 43–48 (2011).

