# Erratum: Spring maize yield, soil water use and water use efficiency under plastic film and straw mulches in the Loess Plateau

**DOI:** 10.1038/srep42455

**Published:** 2017-03-15

**Authors:** Wen Lin, Wenzhao Liu, Qingwu Xue

Scientific Reports 6: Article number: 3899510.1038/srep38995; published online: 12 15 2016; updated: 03 15 2017

This article had a row omitted in Table 1, the correct table is below:

## Figures and Tables

**Table 1:** 

Treatment	Precipitation mm	0–600 cm			0–300 cm		
		CK	SM	PM	CK	SM	PM
2009	357	−27ab	−55a	1b	−26ab	−50a	−2b
2010	543	−144b	−177a	−142b	−143a	−143a	−151a
2011	468	−107a	−115a	−40b	−103a	−126a	−47b
2012	344	32a	49a	60a	20a	18a	44b
2013	400	−69a	−35a	−66a	−78a	−54a	−70a
2014	268	89a	96a	141a	111a	111a	164b
2015	361	105b	113b	64a	93ab	109b	73a
7-yr-change	—	44a	−7a	−9a	51b	22ab	8a

